# Supplementary material for: Antibiotherapy in Children with Cystic Fibrosis—An Extensive Review
Source: Children (Basel). 2022 Aug 20;9(8):1258. doi: 10.3390/children9081258 (PMC9406924; doi:10.3390/children9081258)
Supplement: Supplementary file 1 [file children-09-01258-s001.zip › children-1843040-supplementary.pdf]

Table S1. Characteristics of the articles included in the study

No.	*Title*	*Authors*	*City and Country*	*Year of Publication*	*Type*	*No. of Cases*	*Observation*
1	Genomic Diversity and Antimicrobial Resistance of Haemophilus Colonizing the Airways of Young Children with Cystic Fibrosis	Watts S.C *et al.*	Melbourne, Australia	2021	Original article	147	
2	Clinical Pharmacokinetic and Pharmacodynamic Considerations in the Drug Treatment of Non-Tuberculous Mycobacteria in Cystic Fibrosis	Andrew Burke *et al.*	Brisbane, Australia	2021	Review article	-	
3	Factors influencing the acquisition and eradication of early Pseudomonas aeruginosa infection in cystic fibrosis.	Jackson L. *et al.*	Toronto, Canada	2021	Review article	-	
4	The clinical and microbiological utility of inhaled aztreonam lysine for the treatment of acute pulmonary exacerbations of cystic fibrosis: An open-label randomised crossover study (AZTEC-CF).	Frost F. *et al.*	Liverpool, UK	2021	Original article	12	
5	Antimicrobial activity of ceftazidime/avibactam, ceftolozane/ tazobactam and comparator agents against Pseudomonas	Sader *et al.*	USA	2021	Original article		

	aeruginosa from cystic fibrosis patients					
6.	Optimization of Aztreonam in Combination With Ceftazidime/Avibactam in a Cystic Fibrosis Patient With Chronic Stenotrophomonas maltophilia Pneumonia Using Therapeutic Drug Monitoring: A Case Study	Cowart *et al.*		2021	Case report	1
7	Ceftazidime-avibactam for the treatment of multidrug resistant Burkholderia cepacia complex in a pediatric cystic fibrosis patient	Nguyen *et al.*		2020	Case report	1
8	Antibiotic treatment for nontuberculous mycobacteria lung infection in people with cystic fibrosis.	Waters V. *et al.*	Toronto, Canada	2020	Review article	-
9	Investigation of Stenotrophomonas maltophilia epidemiology in a French cystic fibrosis center.	Capaldo C. *et al.*	Brest, France	2020	Original article	90
10	Phase I, Dose-Escalating Study of the Safety and Pharmacokinetics of Inhaled Dry-Powder Vancomycin (AeroVanc) in Volunteers and Patients with Cystic Fibrosis: a New Approach to Therapy for	Waterer G. *et al.*	Perth, Australia	2020	Clinical Trial	29

	Methicillin-Resistant Staphylococcus aureus.						
11	Antimicrobial Treatment of Staphylococcus aureus in Patients With Cystic Fibrosis	Esposito S. *et al.*	Perugia, Italy	2019	Review article		
12	Methicillin-resistant Staphylococcus aureus eradication in cystic fibrosis patients: A randomized multicenter study.	Dolce D. *et al.*	Florence, Rome, Milan, Messina and Naples, Italy	2019	Original article	61	
13	Eradication of persistent methicillin-resistant Staphylococcus aureus infection in cystic fibrosis	Dezube R. *et al.*	Baltimore, United States	2019	Clinical Trial	29	
14	Interventions for the eradication of meticillin-resistant Staphylococcus aureus (MRSA) in people with cystic fibrosis	Lo DKH *et al.*	Leicester, UK	2018	Review article	106	Searched two clinical trials.
15	Stenotrophomonas maltophilia: A marker of lung disease severity.	Berdah L. *et al.*	Paris, France	2018	Original article	23	
16	Biology and management of methicillin resistant Staphylococcus aureus in cystic fibrosis.	Akil N. *et al.*	Chapel Hill, North Carolina	2018	Review article		
17	Antibiotic strategies for eradicating Pseudomonas aeruginosa in people with cystic fibrosis.	Langton Hewer SC. *Et al.*	Bristol, UK	2017	Review article		
18	Pharmacokinetic-Pharmacodynamic Target Attainment	Bensman *et al.*	USA	2017	Original article	12	

	Analyses To Determine Optimal Dosing of Ceftazidime-Avibactam for the Treatment of Acute Pulmonary Exacerbations in Patients with Cystic Fibrosis					
19	Burkholderia cenocepacia infections in cystic fibrosis patients: Drug resistance and therapeutic approaches.	Scoffone V.C *et al.*	Pavia, Italy	2017	Review article	
20	Evolution of stenotrophomonas maltophilia in cystic fibrosis lung over chronic infection: A genomic and phenotypic population study	Esposito A, *et al.*	Italy	2017	Original article	10
21	Outcomes associated with antibiotic regimens for treatment of Mycobacterium abscessus in cystic fibrosis patients.	DaCosta A, *et al.*	Chapel Hill, North Carolina	2017	Original article	37
22	US Cystic Fibrosis Foundation and European Cystic Fibrosis Society consensus recommendations for the management of non-tuberculous mycobacteria in individuals with cystic fibrosis.	Floto A, *et al.*		2016	Review article	
23	Antibiotic treatment for Stenotrophomonas	Amin R,Waters V.	Toronto, Canada.	2016	Review article	

	maltophilia in people with cystic fibrosis.					
24	Continuous alternating inhaled antibiotics for chronic pseudomonal infection in cystic fibrosis.	Patrick A. Flume *et al.*		2016	Original article	90
25	Risk factors for lung function decline in a large cohort of young cystic fibrosis patients.	Cogen J, *et al.*	United States	2015	Original article	946
26	Inhaled therapy in cystic fibrosis: agents, devices and regimens.	Penny Agent, Helen Parrott	London, UK	2015	Review article	-
27	Infection prevention and control guideline for cystic fibrosis: 2013 update.	Saiman L, *et al.*		2014	Guideline	
28	Antibiotic management of lung infections in cystic fibrosis. II. Nontuberculous mycobacteria, anaerobic bacteria, and fungi.	Chmiel JF, *et al.*		2014	Review	
29	European Cystic Fibrosis Society Standards of Care: Best Practice guidelines.	Smyth AR, *et al.*		2014	Review	
30	Cystic fibrosis foundation pulmonary guideline pharmacologic approaches to prevention and Eradication of Initial Pseudomonas aeruginosa Infection.	Mogayzel PJ, *et al.*		2014	Review	
31	Antibiotic management of lung infections in cystic fibrosis. I. The	Chmiel JF, *et al.*		2014	Review	

	microbiome, methicillin-resistant Staphylococcus aureus, gram-negative bacteria, and multiple infections.					
32	Treatment of lung infection in patients with cystic fibrosis: Current and future strategies.	Döring G, *et al.*		2012	Review	
33	Phenotypes selected during chronic lung infection in cystic fibrosis patients: implications for the treatment of Pseudomonas aeruginosa biofilm infections.	Ciofu O, *et al.*	Copenhagen, Denmark	2012	Original article	
34	Incidence and risk factors for pulmonary exacerbation treatment failures in patients with cystic fibrosis chronically infected with Pseudomonas aeruginosa.	Parkins MD, *et al.*	Northern Ireland	2012	Original article	101
35	Haemophilus influenzae Forms Biofilms on Airway Epithelia.	Starner TD, *et al.*	Iowa	2012	Original article	10
36	Haemophilus influenzae in children with cystic fibrosis: Antimicrobial susceptibility, molecular epidemiology, distribution of adhesins and biofilm formation.	Cardines R, *et al.*	Rome, Italy	2012	Original article	300
37	Therapeutic approaches to chronic cystic fibrosis respiratory infections	Ballmann M, *et al.*		2011	Review	

	with available, emerging aerosolized antibiotics.					
38	Review: Staphylococcus aureus and MRSA in cystic fibrosis.	Goss CH, *et al.*	Seattle WA, United States	2011	Review	
39	Clinical practice guidelines by the Infectious Diseases Society of America for the treatment of methicillin-resistant Staphylococcus aureus infections in adults and children: executive summary.	Liu C, *et al.*		2011	Review	
40	Linezolid vs Glycopeptide Antibiotics for the Treatment of Suspected Methicillin-Resistant Staphylococcus aureus Nosocomial Pneumonia: A Meta-analysis of Randomized Controlled Trials.	Walkey AJ., *et al.*		2011	Meta-analysis	
41	Stenotrophomonas maltophilia in cystic fibrosis: Serologic response and effect on lung disease.	Waters V., *et al.*	Toronto, Ontario, Canada	2011	Original article	692
42	Pseudomonas aeruginosa biofilms in cystic fibrosis.	Høiby N., *et al.*	Copenhagen, Denmark	2010	Review article	
43	An 18-month study of the safety and efficacy of repeated courses of inhaled aztreonam lysine in cystic fibrosis.	Oermann C.M., *et al.*		2010	Original article	274
44	Effect of azithromycin on pulmonary function	Saiman L, *et al.*	New York, United States	2010	Original article	260

	in patients with cystic fibrosis uninfected with Pseudomonas aeruginosa: a randomized controlled trial.					
45	Burkholderia cenocepacia in cystic fibrosis: Epidemiology and molecular mechanisms of virulence.	Drevinek P., *et al.*	Prague, Czech Republic.	2010	Review article	
46	Challenging and emerging pathogens in cystic fibrosis.	De Vrankrijker A.M.M., *et al.*	Utrecht, The Netherlands	2010	Review article	
47	Nontuberculous Mycobacteria–associated Lung Disease in Hospitalized Persons, United States, 1998–2005.	Billinger M.E., *et al.*	United States	2009	Review article	
48	Cystic fibrosis pulmonary guidelines: treatment of pulmonary exacerbations.	Flume P.A., *et al.*	United States	2009	Review article	
49	Early Anti-Pseudomonal Acquisition in Young Patients with Cystic Fibrosis: Rationale and Design of the EPIC Clinical Trial and Observational Study.	Treggiari M.M., *et al.*	Seattle, , United States	2009	Clinical Trial	1787
50	Impact of Pseudomonas and Staphylococcus Infection on Inflammation and Clinical Status in Young Children with Cystic Fibrosis.	Sagel S.D., *et al.*	Colorado, United States.	2009	Original article	111
51	Therapeutic options for Burkholderia cepacia	Avgeri S.G., *et al.*	Athens, Greece.	2009	Review article	

	infections beyond co-trimoxazole: a systematic review of the clinical evidence.					
52	Persistent methicillin-resistant Staphylococcus aureus and rate of FEV1 decline in cystic fibrosis.	Dasenbrook E.C., *et al.*	Baltimore, Maryland, United States	2008	Original article	17.357
53	Achromobacter xylosoxidans in cystic fibrosis: Prevalence and clinical relevance.	De Baets F., *et al.*	Ghent, Belgium	2007	Original article	140
54	The multifarious, multireplicon Burkholderia cepacia complex.	Mahenthiralingam E, *et al.*	Cardiff, UK	2005	Review article	
55	Eradication of early Pseudomonas aeruginosa infection.	Høiby N., *et al.*	Copenhagen, Denmark	2005	Review article	
56	Combination antibiotic susceptibility testing to treat exacerbations of cystic fibrosis associated with multiresistant bacteria: a randomised, double-blind, controlled clinical trial.	Aaron S.D., *et al.*	Ottawa, Canada	2005	Original article	251
57	Multiresistant pulmonary infection in cystic fibrosis—prevention is better than cure.	Smyth A.	Nottingham, UK	2005	Review article	
58	Early intervention and prevention of lung disease in cystic fibrosis: a European consensus.	Döring G., *et al.*		2004	Review article	
59	Infection Control Recommendations for Patients With Cystic Fibrosis: Microbiology,	Saiman L, *et al.*	United States	2003	Review article	

	Important Pathogens, and Infection Control Practices to Prevent Patient-to-Patient Transmission.					
60	Evaluation of a new definition for chronic Pseudomonas aeruginosa infection in cystic fibrosis patients.	Lee T.W.R., *et al.*	Leeds, UK	2003	Original article	232
61	Significant microbiological effect of inhaled tobramycin in young children with cystic fibrosis.	Gibson R.L., *et al.*	United States	2003	Original article	98
62	Susceptibility Testing of Pseudomonas aeruginosa Isolates and Clinical Response to Parenteral Antibiotic Administration Lack of Association in Cystic Fibrosis.	Smith A.L, *et al.*	Seattle, United States	2003	Original article	520
63	Nontuberculous mycobacteria. I: multicenter prevalence study in cystic fibrosis.	Olivier K.N., *et al.*	Chapel Hill, United States	2003	Original article	986
64	Epidemiology of Burkholderia cepacia Complex in Patients with Cystic Fibrosis, Canada.	Speert D.P., *et al.*	Canada	2002	Original article	459
65	Antibiotic prophylaxis in infants and young children with cystic fibrosis: A randomized controlled trial.	Stutman H.R., *et al.*	California, United States	2002	Original article	119
66	Understanding bacterial biofilms in patients with cystic fibrosis: current	Høiby N.	Copenhagen, Denmark	2002	Review article	

	and innovative approaches to potential therapies.					
67	Identification and antimicrobial susceptibility of Alcaligenes xylosoxidans isolated from patients with cystic fibrosis.	Saiman L., *et al.*	New York, United States	2001	Original article	78
68	Serum and lower respiratory tract drug concentrations after tobramycin inhalation in young children with cystic fibrosis.	Rosenfeld M., *et al.*	Washington, United States	2001	Original article	25
69	Effect of continuous antistaphylococcal therapy on the rate of P. aeruginosa acquisition in patients with cystic fibrosis.	Ratjen F., *et al.*	Germany	2001	Original article	639
70	Antibiotic therapy against Pseudomonas aeruginosa in cystic fibrosis: a European consensus.	Döring G., *et al.*		2000	Review	
71	Changing Epidemiology of Pseudomonas aeruginosa Infection in Danish Cystic Fibrosis Patients (1974-1995).	Frederiksen B., *et al.*	Copenhagen, Denmark.	1999	Original article	256
72	Antibiotic susceptibility of multiply resistant Pseudomonas aeruginosa isolated from patients with cystic fibrosis, including candidates for transplantation.	Saiman L, *et al.*	New York, United States	1996	Original article	172

73	Risk factors for Pseudomonas aeruginosa colonization in cystic fibrosis patients.	Kerem E., *et al.*	Toronto, Canada.	1990	Original article	502
74	Haemophilus infection in cystic fibrosis.	Rayner R.J., *et al.*	Nottingham, Uk	1990	Original article	27
